# Calcaneal Ultrasound and Its Relation to Dietary and Lifestyle Factors, Anthropometry, and Vitamin D Deficiency in Young Medical Students

**DOI:** 10.3389/fendo.2020.601562

**Published:** 2021-01-21

**Authors:** Lena Jafri, Hafsa Majid, Sibtain Ahmed, Ghazala Naureen, Aysha Habib Khan

**Affiliations:** ^1^ Section of Chemical Pathology, Department of Pathology and Laboratory Medicine, Aga Khan University, Karachi, Pakistan; ^2^ Department of Medicine - Western Health, The University of Melbourne and Australian Institute for Musculoskeletal Science, The University of Melbourne and Western Health, St Albans, VIC, Australia

**Keywords:** bone, Vitamin D, calcium, lifestyle, body mass index, protein, fat, heel ultrasound

## Abstract

**Background:**

Bone quality and peak bone mass are greatly affected by lifestyle factors. The objective of the study was to investigate the relationships between anthropometry, dietary and caloric intake, body composition measurements, physical activity, and vitamin D status with quantitative ultrasound-based bone parameters among medical students.

**Methods:**

Both male and female medical college students were included in this study. A detailed questionnaire was administered, collecting clinical, dietary, physical activity information, physical examination details, including body mass index (BMI). Body composition (total body fat, total body water, muscle mass, mean visceral fat mass, basal metabolic rate, bone mass using a bioelectrical impedance analyzer) and calcaneal heel ultrasound parameters were measured using an Osteosys Sonost-3000, Ultrasound Bone Densitometer were measured, respectively.

**Results:**

In this cross-sectional study, 211 healthy students with a mean age of 20.1 ± 1.1 years, 51.7% (n = 109) were males. Majority (79.4%) of the young adults had vitamin D deficiency. The mean BMI, calcium intake, and vitamin D levels were 22.35 ± 3.43 kg/m^2^, 788.7 ± 364.8 mg/day, and 15.02 ± 8.63 ng/ml, respectively. Female subjects compared to males had statistically significantly lower daily energy intake, muscle mass, visceral fat mass, calcium intake, and vitamin D levels. In addition the median Z-scores in females [−1.40 (−0.57 to −1.82)] was significantly poorer than the male [−0.50 (0.20 to −1.3)] counterparts, *p*-value <0.001. Multiple regression analysis showed that overall body fat percent (p-value 0.016) and visceral fat percent (p-value 0.029) were the only significant negative predictors to the calcaneal bone quality index (BQI) values.

**Conclusion:**

Adolescent lifestyle patterns can influence young adult bone strength. The young Pakistani females exhibited significantly lower dietary intakes and more inadequate bone parameters compared to males. Our data suggest that total body and visceral fat percent are the predominant negatively associated determinant of bone strength for this cohort. Calcaneal ultrasound can be utilized for mass screening of young adults for identification of low BMD.

## Introduction

Bone mineral status is a function of the maximum bone mineral mass attained in young adult life and of subsequent age-related bone loss. Increasing peak bone mass in young persons could reduce the incidence of later osteoporosis or fragility fracture (1). There are a variety of known determinants of bone mineralization and peak bone mass like genetic makeup, race, weight-bearing physical activity, and hormonal and environmental factors. Some of the factors that increase the risk of osteoporosis in our population are an alarmingly high prevalence of vitamin D deficiency (VDD), small bone frame, and inadequate intake of calcium. Modification of diet and physical activity during a young age may be an effective strategy for maximizing peak bone mass and preventing osteoporosis.

Dual X-ray absorptiometry (DXA) is the gold standard tool for assessing bone mineral density (BMD) in clinical and research practice, but quantitative ultrasound (QUS) measurement of the calcaneus has been suggested as an alternate method in assessing BMD in community as it is relatively inexpensive in contrast to DXA which is also not widely available in lower middle income countries. Moderate correlations have been noted between calcaneal quantitative ultrasound measurements and BMD measurements by DXA in adults (2–4). Screening with heel ultrasound should always be confirmed with BMD by DXA.

In Pakistan, osteoporosis occurs at a relatively earlier age, which has been attributed to lifestyle factors, nutritional deficiencies, and lack of physical activity in young age. Little is known about the influence of diet, body composition, and vitamin D levels on bone mass of Pakistani young adults. Hence we performed this study to determine calcaneal bone mass using the ultrasound technique in apparently healthy adolescents and explore association with anthropometry, body composition, nutrition, life style, and vitamin D in apparently healthy medical students.

## Materials and Methods

### Subjects

A cross-sectional study was conducted on apparently healthy medical students from July 2014 to July 2016 at Aga Khan University (AKU), Karachi, Pakistan. Approval from the AKUH Ethical Review Committee was taken for conducting this study (2810-Pat-ERC-13). A sample size of 165 was calculated by using OpenID, Version 3 software by taking the population size as 100,000 and a hypothesized % frequency of osteopenia and osteoporosis in the population as 50% as shown by Sengodan, Vetrivel Chezian, et al. in evaluation of a cohort of medical and paramedical staffs in a tertiary health-care hospital in India (5). A confidence limit of 80% and a design effect of 1.0 for random sample analysis were used. To ensure maximum power of the study, 211 subjects were recruited based on consecutive sampling until time and date saturation of the study design, i.e. significantly higher than the anticipated sample size calculated.

A 4-day camp was held at the university campus for collecting clinical, dietary, physical activity information, physical examination, and phlebotomy. The protocol and objectives of the study were explained to the students, and written consent was taken by trained personnel. All recruits were healthy, meaning did not have any known or diagnosed disease, had not been diagnosed with a genetic or metabolic disorder at the time of sample collection, and were not on any medications as per subjects’ recall. While students who had vitamin D injection in the last 6 months or had remarkable changes in weight or diet during the previous 6 months or any disease/metabolic disorder that may affect their vitamin D levels were excluded. Each participant was allotted an identifying serial number. Body composition measurements done were total body fat, total body water, muscle mass, mean visceral fat mass, physical rating, basal metabolic rate (BMR), bone mass of the participants using a bioelectrical impedance analyzer (BIA). Also, calcaneal ultrasound measurements were done, including bone quality index (BQI), speed of sound (SOS), and broadband ultrasound attenuation (BUA) for two consecutive batches of second-year medical students.

### Medical History, Sociodemographic Factors, and Dietary Assessment

An interviewer-administered questionnaire tailored for the adult Pakistani urban population was used to evaluate the clinical history and health status of the study subjects. Socio-demographic factors, type of residence (apartment/hostel, independent small houses, or large independent bungalow with garden or open space) of students were assessed. Dietary data were collected with specially designed semi-quantitative validated food frequency questionnaire (FFQ) (6). Available frequency of food consumption ranged from “once a month” to “once or more than once a day. The portion sizes used in the FFQ were based on a typical or natural portion (e.g., slice/piece, cup, bowl, etc.). This information was used for assessing the mean intake of calcium, protein, fat, and energy of the participants through a face-to-face interview by trained personnel. Observed intakes were compared to the recommended daily allowance (RDA) according to age and gender.

### Anthropometric and Bioelectrical Impedance Analyzer Measurements

All of the anthropometric measurements were done by trained personnel Anthropometric characteristics (height, weight, and waist circumference) and body composition, i.e., fat percentage, muscle mass; bone mass, BMR physical rating, visceral fat, and metabolic age, of the participants (using bioelectrical impedance analysis scale) were measured once only. A “bioelectrical impedance analyzer” by Tanita (Tanita Corporation, Arlington Heights, IL, USA) in bare‐footed participants wearing light clothing was used for body composition assessment of the subjects. BIA is a simple validated noninvasive method of assessing body composition in both clinical and non-clinical settings.

Overeating, overdrinking, heavy sweating during vigorous exercise, or alcohol consumption or the use of diuretics could all lead to inaccuracy (1–2% imprecision) in BIA recordings. To ensure reliable stable BIA readings it was ensured that 3 h have passed after getting up and normal lifestyle activities of subjects were carried out during this period before taking measurements (7). Smoking and alcohol consumption were made part of exclusion criteria. The BIA measurements included total body fat percentage, total body water percentage, total muscle mass, basal metabolic rate, and total bone mass. International body fat reference ranges for young adults were used to categorize the subjects. BMI was calculated as body weight in kilograms divided by square of height in meter. BMI values were classified as per the recommended reference ranges for the WHO South Asian Classification (8).

### Blood Collection and Biochemical Analysis

Blood was drawn in gel separation tubes and centrifuged for separation of serum. Separated serum was transferred to aliquots, labeled with the participant’s serial number, and immediately stored at −30°C. Serum 25-hydroxy vitamin D (25OHD) levels of all study participants were measured by a chemiluminescence assay on ADVIA Centaur Total vitamin D Assay from Siemens Medical Solutions Diagnostics, Tarrytown, and NY, USA. The analytical measuring range of the 25OHD assay was 4.20–150.0 ng/ml with 4.2–11.9% coefficient of variation. Serum samples were run in batches with three level internal quality controls to validate the 25OHD results. Vitamin D deficiency, insufficiency, and sufficiency were defined as <20 ng/ml, 20–30 ng/ml, and > 30 ng/ml respectively.

### Bone Mineral Density Measurements

Osteosys Sonost-3000, Ultrasound Bone Densitometer was used to measure right side calcaneal ultrasound of study participants. It was calibrated and precision (manufacturer claim of coefficient of variation being <1%) was measured before usage to reduce a margin of error and to get accurate value when measuring calcaneal bone density. The measurements were obtained once from the right calcaneus of all students in a room temperature controlled environment, as suggested by the manufacturer. All measurements were taken by the same investigator throughout the study. Measurements provided were Z-score, Bone Quality index (BQI), Speed of Sound (SOS), and BUA (broadband ultrasound attenuation). The Z-scores were generated by the Ultrasound Bone Densitometer by comparing BQI with age specific Korean reference population. To define low bone mass Z-score cutoff of −2 was used (1). Bone quality was expressed using the BQI, calculated by the device from the QUS parameters SOS and BUA, indicating bone structure. BUA (dB/MHz) is the attenuation of sound waves as they pass from the transmitting transducer to the receiving transducer. The SOS (m/s) is the speed the ultrasound signal travels from one transducer to the other. Normal bone has a higher BUA and SOS than osteoporotic bone.

### Statistical Analysis

Data were coded, and all statistical analysis was done on the Statistical Package for Social Sciences (Version 22, SPSS Inc. Chicago, IL, USA). All parametric variables were reported as mean ± S.D. and non-parametric as median with interquartile ranges 75^th^–25^th^. Differences between two groups of parametric variables were evaluated using independent samples t-test. To compare median values between two or more groups, the Mann Whitney U test and the Kruskal-Wallis test were applied respectively. Association of study variables with BQI were calculated using Spearman’s correlation coefficient. Univariate analysis was conducted, and variables that had a significant association with BQI in a univariate analysis at P < 0.2 were entered in multivariable analysis in a stepwise manner. Multivariate regression analyses were conducted for the dependent variable (BQI) and independent variables (lifestyle and dietary factors, body composition, and 25OHD). For working out the magnitude of associations between independent-dependent variables, the beta weight of the variables was calculated. The level of significance was set to *p-*value less than 0.05 as significant, and <0.001 was considered highly significant.

## Results

### Description of Subjects

Two hundred and eleven students (109 males and 102 females) were enrolled. The mean age was 20.1 ± 1.1 years, 51.7% (n = 109) being males. **Table 1** shows the socio-demographic details of the students overall and according to gender. None of the students had history of alcohol consumption, medications, or supplementations whereas 9% (n = 19) gave a history of cigarette smoking. Majority (81%, n = 171) lived in apartments or hostels, 5.7% (n = 12) in independent small houses, and 13.3% (n = 28) in independent large bungalows.

**Table 1:** Body composition, dietary intake, lifestyle factors, and bone mineral density of medical students (n = 211).

Variables	All Subjects n = 211	Males n = 109	Females n = 102		*p* value	
Anthropometric						
Mean Age (Years)	20.15 ± 1.18	20.37 ± 1.24	19.92 ± 1.07		0.006	
Mean Height (cm)	168.59 ± 9.42	174.74 ± 7.29	162 ± 0.02		<0.001	
Mean Weight (kg)	63.80 ± 12.80	70.13 ± 11.60	57.20 ± 10.66		<0.001	
Mean Waist Circumference (cm)	77.07 ± 9.10	81.80 ± 7.28	72.02 ± 8.27		<0.001	
Mean BMI (kg/m^2^)	22.35 ± 3.43	22.85 ± 3.17	21.82 ± 3.63		0.029	
Dietary daily intake of calcium, proteins, carbohydrates, fats, and energy						
Mean calcium intake (mg)	788.7 ± 364.8	937.6 ± 364.7	629.7 ± 291.7	<0.001	
Mean protein intake (g)	66.4 ± 30.8	75.7 ± 34.5	56.4 ± 22	<0.001	
Mean fat intake (g)	63.7 ± 28.2	72.9 ± 29.5	53.9 ± 23.2	<0.001	
Mean energy intake (kcal)	2,210.4 ± 891.6	2,619.9 ± 849.2	1,772.8 ± 712.4	<0.001	
Body composition						
Mean Total Body Fat %	18.3 ± 8.65	13.44 ± 6.02	23.60 ± 7.92		<0.001	
Mean Total Body Water %	53.97 ± 6.69	50.65 ± 7.02	57.08 ± 4.55		<0.001	
Mean Muscle Mass (kg)	41.28 ± 7.45	45.71 ± 5.97	36.54 ± 5.79		<0.001	
Mean Visceral Fat Mass (kg)	1.86 ± 1.52	2.38 ± 1.90	1.48 ± 1.05		0.96	
Mean Physical Rating	4.29 ± 1.85	4.20 ± 1.31	4.29 ± 2.20		0.45	
Mean Basal Metabolic Rate (kcal)	1301.9 ± 171.8	1315.8 ± 180.6	1287.1 ± 161.6		0.22	
Mean Bone Mass (kg)	2.51 ± 0.47	2.86 ± 0.34	2.13 ± 0.25		<0.001	
BMD parameters						
Median BQI	85.4 (98.7 to 74.2)	93.40 (102.3–79.9)	77.85 (90.25–70.8)		<0.001	
Median SOS (m/s)	1,521 (1,531.6 to 1,510.6)	1,527.00 (1,533.8 to 1,516.1)	1,515.25 (1,524.5 to 1,508.3)		0.002	
Median BUA (dB/MHz)	86.4 (103.7 to 70.3)	92.3 (107 to 80.7)	80.6 (96.0 to 64.6)		<0.001	
Median T-Scores	−1.00 (−0.20 to −1.60)	−0.60 (−0.0 to −1.30)	−1.30 (−0.67 to −1.70)		<0.001	
Median Z-Score	−0.90 (−0.10 to −1.70)	−0.50 (0.20 to −1.3)	−1.40 (−0.57 to −1.82)		<0.001	
Biochemical parameters						
Median serum 25OHD (ng/ml)	14.3 (16.7–9.4)	15.23 (18.95–12.3)	12.01 (18.54–8.5)		0.002	

Student t test and Kruskal Wallis were applied to study the mean and median comparison between genders respectively.

BMI, Body Mass Index; BMD, Bone Mineral Density; BQI, Bone Quality Index; SOS, Speed of Sound; BUA, Broadband Ultrasound Attenuation; 25OHD, 25-hydroxy vitamin D.

### Anthropometry and Bioelectrical Impedance Analyzer Analysis

Male adolescents had significantly higher body height and weight than females (*p*-value <0.001).The mean BMI was 22.35 ± 3.43 kg/m^2^. In accordance with the South Asian Classification of weight status, 11.8% of the subjects were categorized as underweight (<18.5 kg/m2), 47.9% as normal (18.5–22.9 kg/m^2^), 20.4% as overweight (23–25 kg/m^2^), and 19.9% as obese (>25 kg/m^2^). **Table 2** compares the distribution of BMI classification as per gender. No statistical difference in BMI between the two genders was noted; however, the mean BMI of males was higher as compared to females. High physical activity was observed in 28% (n = 59) of the students, while moderate and low physical activity was observed in 46.4% (n = 98) and 25.6% (n = 54) of students, respectively.

**Table 2:** Descriptive categorization of subjects according to dietary intake, bioelectrical impedance analysis (BIA), biochemical and calcaneal ultrasound findings.

Study variables		Categories	Male (n = 109)		Female (n = 102)		
			Reference range or cutoff	n (%)	Reference range or cutoff	n (%)
Dietary intake	Calcium intake (mg/day)	Below RDA	<1,000	73 (67)	<1,000	90 (88.2)
	Protein intake (g/day)	Below RDA	<52	32 (29.4)	<46	40 (39.2)
BIA	Body fat range (%)	Under fat	0–10	40 (36.7)	0–20	37 (36.3)
		Standard minus	11–16	44 (40.4)	21–27	35 (34.3)
		Standard plus	17–21	12 (11)	28-34	21 (20.6)
		Over fat	21–25	8 (8.3)	35–39	6 (5.9)
		Obese	26–45	4 (3.7)	40–45	3 (2.9)
	Visceral fat rating	Standard	<9	109 (100)	<9	102 (100)
		High	10–14	–	10–14	–
		Very high	>15	–	>15	–
	Physique rating	Hidden obese	1	–	1	10 (9.8)
		Obese	2	13 (11.9)	2	16 (15.7)
		Solidly built	3	23 (21.1)	3	16 (15.7)
		Under exercised	4	19 (17.4)	4	8 (7.8)
		Standard	5	43 (39.4)	5	28 (27.5)
		Standard muscular	6	5 (4.6)	6	2 (2)
		Thin	7	6 (5.5)	7	4 (3.9)
		Thin and Muscular	8	–	8	16 (15.7)
		Very Muscular	9	–	9	2 (2)
	BMI (kg/m^2^)	Underweight	<18.5 kg/m^2^	9 (8.3)	<18.5	16 (15.7)
		Normal	18.5–22.9 kg/m^2^	48 (44)	18.5–25	53 (52)
		Overweight	23–25 kg/m^2^	27 (24.8)	25–30	16 (15.7)
		Obese	>25 kg/m^2^	25 (22.9)	>30	17 (16.7)
	Bone mass (kg)	Low bone mass	<1.8 kg for weight <60 kg	1 (0.9)	<1.8 kg for weight <45 kg	4 (3.9)
			<2.2 kg for weight 60–75 kg	3 (2.8)	<2.2 kg for weight 45–60 kg	42 (41.2)
			<2.5 kg for weight >75 kg	–	<2.5 kg for weight >60 kg	16 (15.7)
Biochemical parameters	Serum 25OHD levels (ng/ml)	Normal	>30	9 (8.3)	>30	12 (11.8)
		Vitamin D insufficiency	20–30	14 (12.8)	20–30	9 (8.8)
		Vitamin D deficiency	<20	86 (78.9)	<20	81 (79.4)
Calcaneal ultrasound	Z-scores	Normal	<−2	102 (93.6)	<−2	82 (80.4)
		Low bone mass	>−2	7 (6.4)	>−2	20 (19.6)

BMI, Body Mass Index; 25OHD, 25-hydroxy vitamin D.

### Dietary Characteristics

Summary results of daily dietary intake as per FFQ analysis according to gender and age are presented in **Table 1**. Female students showed lower mean daily energy intake (*p*-value <0.001), mean muscle mass (*p*-value <0.001), and mean visceral fat mass (*p*-value 0.003) as compared to the males. Daily consumed amounts of calcium, proteins, fat, and energy was significantly higher in males than females (<0.001). In 163 students (77.2%), the average calcium intake was below Recommended Dietary Allowance (RDA) (1,000 mg/day for 19–50 years) (9). Out of the total 65.8% (n = 139) students, the protein intake was higher than the RDA in both age groups, and genders.

### Vitamin D Status

Mean 25OHD level was 15.02 ± 8.63 ng/ml with 79.1% (n = 167) found to be deficient in 25OHD levels, 10.9% (n = 23) had insufficient serum 25OHD levels, and 10% (n = 21) had sufficient levels. Median 25OHD levels were higher in male counterparts (15.23 ng/ml) as compared to females (12.01 ng/ml); p value <0.05. Median 25OHD in students with low, moderate, and high physical activity were 12.01 (19.45–9.0), 14.22 (18.62–9.22), and 15.94 (18.67–12.5) ng/ml, respectively. The difference in median 25OHD levels across different physical activity categories was statistically significant (*p*-value 0.012).

Median 25OHD in students living in apartments/hostels, independent small houses and in independent large bungalows were 14.0 (18.01–9.23), 18.15 (23.75–11.07), and 17.33 (26.65–12.05) ng/ml respectively. This difference in median 25OHD levels in students living in different types of residence was statistically significant (*p*-value 0.018) with highest median 25OHD noted in students living in independent small houses.

### Bone Mineral Status

All the parameters of quantitative calcaneal ultrasound bone measurements (BQI, BUA, and SOS) are described in **Tables 1** and **2**. The males had significantly higher BQI than the females; *p-*value <0.001. Based on Z-scores, out of the total 211 students, 12.8% (n = 27) students were observed to have low bone mass (Z score <−2 SD). The median Z-scores in females [−1.40 (−0.57 to −1.82)] was significantly poorer than the male [−0.50 (0.20 to −1.3)] counterparts, *p*-value <0.001. Bone quality index (BQI) and Z-score were not significantly different between those who consumed more or less than 1,000 mg of calcium; *p*-value 0.568 and 0.882, respectively. Median BQI in students living in small apartments/hostels, independent small houses, and in independent large bungalows were 82.4 (97.4–82.4), 83.35 (101.5–75.6), and 92.2 (104.4–82.5) respectively. This difference in median 25OHD levels in students living in different types of residence was statistically significant (*p*-value 0.029).

### Correlates of Bone Quality Index and Multiple Regression Analysis

**Table 3** describes the relevant Spearman correlation coefﬁcients and their degrees of signiﬁcance between BQI and other variables in the total study population and both genders separately. In the overall group (n = 211) significant positive correlation was seen between BQI and BMI, daily calcium intake and daily energy intake, while significant inverse relation was seen with total body fat % and physique rating. In males, BQI exhibited inverse relation with muscle mass (p-value <0.05) and physical rating (p-value <0.05). On the other hand in females BQI exhibited positive relation with BMI (p-value <0.05) and visceral fat % (p-value <0.05), while inverse relation with BMR (p-value <0.001), body water % (p-value <0.001), and physique rating (p-value <0.001). Variables that had a significant association with BQI univariate analysis at P < 0.2 were entered in multivariable analysis.

**Table 3:** Correlation of lifestyle factors, biochemical parameters, anthropometric and BIA measurements with BQI according to gender distribution.

Gender	n	Age	BMI	Calcium intake (mg)	Protein intake (g)	Fat intake (g)	Energy intake (kcal)	Total body fat %	Total body water %	Muscle mass (kg)	Visceral fat %	Physique rating	Basal metabolic rate (kcal)	Z-scores	Serum 25OHD levels (ng/ml)
Male	109	−0.002	0.13	−0.01	−0.08	−0.02	0.11	0.04	−0.11	−0.24*	−0.02	−0.2*	−-0.13	0.94**	−0.09
Female	102	−0.05	0.23*	0.07	0.09	−0.02	0.13	0.17	−0.41**	−0.27**	0.23*	−0.40**	−0.33**	0.88**	−0.01
Overall	211	0.032	0.24**	0.2**	0.08	0.08	0.30**	−0.15*	0.004	0.07	0.11	−0.27**	0.09	0.91**	0.04

*Correlation is significant at the 0.05 level (two-tailed) and **highly significant at the 0.01 level (two-tailed).

Multiple regression analysis showed that body fat percent and visceral fat percent were the only contributing factors (negative predictors) to BQI values (**Table 4**). The value of R square and adjusted R square was 0.674 and 0.659 respectively specifying moderate association between various independent and dependent variables.

**Table 4:** Multiple regression analysis of BQI determinants.

Independent variables	All participants		Male		Female	
	B	*P* value	β	*P* value	β	*P* value
BMI	0.09	0.114	0.109	0.236	0.103	0.380
TCA	−0.03	0.655	−0.055	0.573	0.025	0.812
TFT	−0.028	0.659	−0.30	0.731	0.012	0.906
TEN	0.159	0.5	−0.030	0.731	0.031	0.792
Body Fat %	−0.033	0.016	=0.026	0.788	−0.037	0.731
Visceral Fat	−0.145	0.007	−0.154	0.046	−0.129	0.197
Physical Rating	0.013	0.787	0.047	0.493	−0.055	0.53
BMR	0.032	0.481	0.052	0.437	−0.003	0.97

## Discussion

This cross-sectional study on 211 apparently healthy medical students revealed that majority (79.4%) had vitamin D deficiency. The young Pakistani females exhibited significantly lower dietary intakes and more inadequate bone parameters on calcaneal ultrasound compared to males. The bone quality index BQI correlated significantly with BMI, calcium intake, energy intake, total body fat %, physical rating, and Z-scores. Out of the total students, 12.8% (n = 27) students were observed to have poor bone density (Z scores less than minus two). Calcaneal QUS is a convenient device for screening for osteoporosis. Literature confirms that the DXA measurements significantly match with calcaneal bone QUS findings (10, 11). QUS has been widely adopted as alternative to DXA for an indirect assessment of bone quality. It offers certain advantages as it is portable, inexpensive, noninvasive with no use of ionizing radiation, in addition to comparable cost and time saving compared to DXA (12). Several studies have indicated that calcaneal bone QUS indices were able to predict fractures in both genders (13, 14). Although several studies and reviews linking nutrition to bone health in young adults have been investigated elsewhere, the research conducted in Pakistan has been limited. Sufficient vitamin D levels are required for calcium absorption and utilization, and for mineral metabolism for bone development and health during young age.

Literature from various parts of the world suggests that VDD is common, both among adults and adolescents (15, 16). The majority of the population residing in Pakistan has VDD (17, 18). Little is known about the prevalence of VDD in healthy young adults. As far as is known, no study has been performed to date on the Pakistani adolescent population to address the influence of diet, body composition, and vitamin D levels on the bone mass. We observed that 79.1% of young adults aged 17 to 24 years had VDD, and most of the students (77.7%) were unable to meet the RDA for calcium. The high prevalence of VDD in young adults may be explained by their inadequate consumption of vitamin D rich foods such as fortified cereals/milk and oily fish, however this was not assessed (19–22). Prevention or control of VDD in young adults can be achieved by lifestyle modification, and encouraging increased vitamin D intake through multivitamin supplementation, and vitamin D fortified food products. The dilemma is that unlike the west, Pakistan does not have a mandatory vitamin D fortification policy in place. Reports have shown that body composition, especially obesity, also increases the prevalence of VDD, and the prevalence of obesity in Pakistan is also reportedly higher (23, 24). Anthropometry and body composition are the measurements of certain parameters of the human body and are frequently used to assess nutritional status as well as growth and development of children and adolescents. Many developing countries including Pakistan face an increasing dual burden of both under and over nutrition (25).

In the current study female subjects compared to males had significantly lower daily energy intake (*p*-value <0.001), muscle mass (*p*-value <0.001), visceral fat mass (*p*-value 0.003), calcium intake (p-value <0.001), and vitamin D levels (p-value <0.05). Adolescents are at an age that is targeted for health messages and in whom significant bone mineral accretion occurs. Mean BMI of the study group was 22.35 ± 3.43 kg/m2 and as per South Asian classification of weight status, 11.8% of the subjects in the current study were categorized as underweight, while 20.4 and 19.9% as overweight and obese respectively. The subjects in this study had higher BMI compared to medical students from Delhi in India and Japan, but showed comparative values to other similar studies reported from Pakistan and another study from Haryana in India (26–29).

In the present study, we found that calcaneal QUS based bone BQI positively correlated with BMI, calcium intake, energy intake, and Z-scores, while negatively correlated with physical rating and total body fat percentage. Multiple regression analysis showed that overall body fat percent (p-value 0.016) and visceral fat percent (p-value 0.029) were the only significant negative predictors to BQI values. The inverse relationship between fat mass and BQI which was statistically significant (p-value 0.016) can be explained through the smaller bone area with increasing fat mass, whereas no evidence for increased bone loss in this young group of medical students could be found. Somewhat similar findings were reported by Sioen I et al., examined 7,447 children (age range 2.1–9.9 years) and stated that body fat was negatively associated with calcaneal stiffness index in children. This study also concluded that muscle mass could be an essential predictor of calcaneal bone stiffness index (30). Our findings are also in conjunction with Takahata Y et al., who reported a strong negative correlation between body fat percentage and calcaneal QUS in a cohort of Japanese undergraduate female students (31). However, some dissimilar findings were reported by Xu Yi et al. in a study done on 392 children (5–19 years) in which calcaneal stiffness index showed significant positive correlations with age, weight, height, BMI, bone mineral density and content, total body lean and fat mass (32). Another study conducted on health Saudi Arabian children and adolescents reported a directly proportional relationship between QUS and physical activity, diet, sun exposure, and calcium supplement intake (33). Recent publications describe direct and indirect effects of adipose tissue on bone. The underlying mechanisms whereby fat can influence bone mass need to be further investigated. Adipocytes, myoblasts, and osteoblasts are all from the same mesenchymal stem cells. Is it during the development that opposing effects on bone and fat occur because of their shared molecular pathways (34)? The fat cells are metabolically active, and both direct and indirect effects of leptin on bone have been reported from *in vitro* and *in vivo* studies (35–37). These unfavorable effects of fat mass on bone mass need to be further This is one of the few studies that address bone health status of young adults from Pakistan. However, there are several limitations of the current study. The sampled population did not represent Pakistani youth in general as the medical students were from a single institution, conveniently sampled. More extensive community-based studies with a larger sample size in the Pakistani youth are required for the confirmation of our findings. The cross-sectional nature of this study could not elucidate causal relationships. However, it outlines an essential interplay between circulating 25OHD and dietary patterns, nutrient and energy intake, and body composition. The majority of our study subjects were deficient in vitamin D and we were not able to measure sun exposure in our study hence adding to the study limitations. Recall bias during data collection could add to current study bias. Hence in planning of similar studies in future biochemical analysis of metabolic disorders should be added in methodology along with review of medical charts of every subject. Additionally the tools used in the study (BIA and ultrasonometer) were not validated in Pakistani population earlier. The Z-score computed by calcaneal ultrasound machine were generated by comparing BQI values with age specific Korean reference population and not Pakistani population. Further studies that build upon our findings and control for possible confounders could be of clinical importance.

In conclusion, our findings showed that female subjects have significantly lower daily energy and calcium intake, muscle mass, visceral fat mass, and vitamin D levels compared to males. The QUS based calcaneal bone BQI correlated significantly with BMI, total body fat %, and visceral fat percent for adolescents aged 19–22 years. Calcaneal QUS can be used for mass screening of adolescence in a country like ours where DXA facilities are scarce.

## Data Availability Statement

The raw data supporting the conclusions of this article will be made available by the authors, without undue reservation.

## Ethics Statement

The studies involving human participants were reviewed and approved by the Ethical Review Committee of Aga Khan University. The patients/participants provided their written informed consent to participate in this study.

## Author Contributions

Here are the most important contributions of each author: AK and LJ designed the study. Data were collected by GN, HM, and SA. Analysis of data was carried out by LJ and HM. All authors take final responsibility for this article. All authors contributed to the article and approved the submitted version.

## Funding

This work was supported by funding from the Medical College of Aga Khan University during the Research Module in the second year of MBBS.

## Conflict of Interest

The authors declare that the research was conducted in the absence of any commercial or financial relationships that could be construed as a potential conflict of interest.
